# Physical and motivational effects of Exergames in healthy adults—A scoping review

**DOI:** 10.1371/journal.pone.0312287

**Published:** 2025-02-07

**Authors:** Katrin Hoffmann, Josef Wiemeyer, Anna Lisa Martin-Niedecken

**Affiliations:** 1 Institute of Sport Science, Technical University Darmstadt, Darmstadt, Germany; 2 Institute for Design Research, Zurich University of the Arts, Zurich, Switzerland; Instituto Politecnico de Santarem Escola Superior de Desporto de Rio Maior, PORTUGAL

## Abstract

Exergames have the potential to be used as physical-cognitive training tools. Although numerous studies investigated the physical and motivational effects of Exergaming, a comprehensive summary of training effects in healthy adults is still missing. This scoping review aims to identify available evidence and research gaps on absolute and relative effects of Exergame training on physical indicators (effectiveness) and motivation (attractiveness) compared to no or conventional training in healthy adults. A systematic literature search was performed in EBSCOhost, WoS CC, SURF, and Science Research for studies meeting the following criteria: a) randomized controlled trials with Exergames compared to conventional training and/ or no treatment; b) healthy adults (age: 18 to 64 years); c) Exergame training interventions; d) assessment of pre-post differences, primary outcomes: endurance, strength, flexibility, speed, balance, sensorimotor coordination, sports skills, player experience; secondary outcomes: adverse events, physical activity level, attitudes, and knowledge. Methodological quality of included studies was assessed using the PEDro Scale. Charted data were categorized according to the PICOS framework. Mean differences, 95% confidence intervals, and effect sizes (ES) were calculated for continuous outcomes. Evidence Maps were created to illustrate the estimated Risk of Bias, estimated effect, and sample sizes. Eighteen publications with 907 participants were included. The number of studies per outcome was low (2 to 9balance:). Studies on coordination and knowledge were lacking. No study analyzed the relative effects of speed training with Exergames. Absolute effects of Exergame training ranged from no to large effects. Significant ES were consistently found for skill training. Negative effects were found for individual parameters. Relative effects of Exergame training ranged from no to large effects (one exception). A moderate absolute effect favoring traditional training was found for skill training. The remaining results suggest that Exergaming elicited similar training effects compared to conventional training. A high variance of applied training, corresponding training parameters (FITT-VP), outcome measures, as well as methodological deficits and incomplete reporting were identified in included studies. Therefore, a lack of high-quality studies and reviews was identified. Future research should conduct high quality RCT studies analyzing the effectiveness and attractiveness in Exergames. In addition, more suitable, well-designed Exergames should be developed.

## Introduction

Regular physical activity (PA) has numerous health benefits for humans of all ages. Health benefits comprise mental effects such as increased quality of live [1] and biological effects, for example on cardiovascular and immune functions [2]. On the other hand, the negative effects of lack of PA or sedentary behavior have also been investigated. For example, physical inactivity has been identified as the fourth leading risk factor for global mortality [3].

However, to elicit sustainable health benefits of PA, several conditions have to be fulfilled. First, physical training has to be appropriate regarding the intended health effects (effectiveness). To ensure effectiveness, for example, WHO [4] and ACSM [5] have published recommendations and guidelines for physical training. Recommendations include endurance, resistance, flexibility, and neuro-motor exercises. One important framework for physical training is the FITT-VP approach comprising frequency, intensity, time, type, volume, and periodization of PA [6]. In particular, the volume of PA has been addressed by the WHO. For example, for adults (age: 18 to 64 years) average weekly volumes of 150 to 300 minutes (moderate intensity) or 75–150 min (vigorous intensity) are recommended [4]. Second, in order to support sustainable engagement in PA, socio-psychological factors have to be considered, for example, emotions, motivation and volition (attractiveness). In this regard, numerous taxonomies, e.g., 99 behavior change methods based on intervention mapping taxonomy [7], as well as specific and unspecific procedural and structural models have been developed, e.g., theory of planned behavior and reasoned action [8], self-determination theory [9], or MoVo approach [10].

Although there is convincing evidence regarding the health benefits of regular PA, physical inactivity and sedentary behavior are a phenomenon that is widespread and has even increased in (high income) Western countries and Latin America [1, 3]. In 2016, overall percentage of physical inactivity of adults was 27.5% [11]. Furthermore, a survey performed in 2022 revealed that even 62% of Europeans reported insufficient levels of physical activity [12]. Among numerous possible causes, motivation plays an important role [13].

In order to achieve both effectiveness and attractiveness of PA, Exergames offer valuable options and a promising approach to implement sustainable behavioral change techniques and thus to overcome potential motivational and emotional barriers. Exergames or Active Video Games (AVG) are a specific type of Serious Games, that require the player to engage in PA including upper or lower body movement to control the game [14]. Exergames aim to provide an entertaining and motivating physical training. An optimal balance of effectiveness and attractiveness [15, 16] is the main challenge of Exergames.

In order to establish this balance, the individual adjustment of training load is very important, as balancing task difficulty and performance level increases the motivation and the adherence to the game [17]. This balance makes the exercises performed more enjoyable, which in turn increases physical self-efficacy and reduces potential inhibitors for future participation in PA. Additionally, playing Exergames transforms a sedentary computer game play to a healthier game activity [18].

Due to this promising potential, Exergames have gained increasing attention in scientific research in the last decades. Currently, numerous studies investigating Exergames are available. However, a recent overview of reviews revealed that a comprehensive summary of conditioning, coordinative or sports skill related physical training effects and motivational effects of Exergames in healthy adults is still missing [19]. Current reviews focus either on specific samples (e.g., children and adolescents, older adults with or without clinical issues [20, 21]) or on selected aspects of physical exercise (e.g., short-term changes in energy expenditure induced by Exergames [22–24]). In particular, reviews focusing on training effects regarding balance, strength, or motor skill training in healthy adults were not found. Furthermore, reviews addressing the analysis of the combination of effectiveness and attractiveness in Exergames are rare. Therefore, a substantial lack of reviews addressing the broad effects of training with Exergames or AVG in healthy adults was identified.

According to the published protocol [25], a systematic review was planned to close this gap and to identify possible moderators and mediators of training effects elicited by Exergames. However, the systematic literature search revealed that the number of suitable studies per outcome was very low and included varying characteristics. As no comprehensive conclusion could be drawn from the available evidence, a systematic review was deemed inappropriate [26, 27]. A scoping review including evidence mapping was performed instead [28].

According to the purposes of scoping reviews, this review aims to:

- identify available evidence for absolute and relative effects of training with Exergames on physical indicators (effectiveness) and motivation (attractiveness) compared to no or conventional training in healthy adults aged between 18 and 64 years;
- identify gaps in the current evidence related to Exergame-based training.

## Methods

This review was conducted according to the published protocol [25] (S1 Protocol) following Cochrane guidelines of reviews [29]. All deviations from the protocol are reported in the section *Differences between protocol and review* of this review. The findings of this research were reported according to the Preferred Reporting Items for Systematic Reviews and Meta-Analyses extension for Scoping Reviews (PRISMA-ScR) guidelines [30].

### Eligibility criteria

Inclusion and exclusion criteria for studies to be considered for this review can be found in Table 1.

**Table 1:** Inclusion and exclusion criteria for studies included in this review.

	Inclusion criteria	Exclusion criteria	Justification of the selected criteria
Types of studies	Randomized controlled trials—no restriction regarding type of randomization	Non-empirical papers using models or opinions as primary source of evidence, case studies, qualitative studies [31]	Ensures high study quality
Types of participants	healthy adults (aged from 18–64 years) [4]; no restriction regarding training status (e.g. trained, sedentary), gender, ethnicity, geographical location, specific racial or cultural interests	studies targeting participants with diseases (e.g., cardiovascular, neural or mental disorders), age below 18 years, age above 64 years	Specifies target group—relevant lack of evidence regarding the included target group
Types of interventions	training interventions performed with Exergames or AVGs; training interventions with Exergames supplemented by additional components without conflicting or confounding influence (e.g., knowledge transfer)	home-based training (possible monitoring issues)	Relevant lack of evidence regarding training with Exergames; ensures high quality of evidence (control issues)
Types of comparators	interventions with control groups: conventional physical training (active control; relative effects) or no training (passive control; absolute effects)	interventions without control groups	Ensures high quality of evidence
Types of outcomes	only pre-post differences comparing baseline to post-intervention performance • primary outcomes [15, 32–34]: endurance or aerobic capacity, strength or resistance, flexibility, speed or agility, balance, complex motor reactions or sensorimotor coordination, sports skills, player Experience • secondary outcomes [15]: adverse events, PA level, attitudes, knowledge		Relevant lack of evidence regarding the specified outcomes
Types of measures	reliable and valid measures of physical training effects [33–37] (physiological/ biomechanical parameters, testing routines, and questionnaires)	Unreliable and invalid measures of physical training effects	Ensures high quality of evidence

### Systematic literature search

An electronic search was performed on May 6^th^ and May 7^th^ 2022 and updated on September 4^th^ 2023. The search was conducted in EBSCOhost, the Web of Science Core Collection, SURF, and Science Research. Due to server errors in the first search, Science Research was only included in the updated search. The advanced search in Science Research comprised the topics “Computer & Technology”, “Health & Medicine”, and “Multidisciplinary Sources”. This includes a variety of databases (i.e., Springer, BioMed Central, Mednar, PubMed). A complete list of databases included by the search engines can be found in the S1 Text. All databases and research platforms were searched using the specific search term in all fields:

(Exergam* OR “Active Video Gam”*) AND (endurance OR aerobic* OR stamina OR strength OR resistance OR flexibility OR agility OR speed OR balance OR “complex motor reaction”* OR “sensorimotor coordination” OR “sport skill”* OR motivation)

Only studies published under peer-review conditions were considered without restriction regarding year of publication to ensure an inclusion of all relevant high-quality studies. Only literature published in German or English was considered.

In addition, a manual search analyzing the reference lists of selected studies was performed to identify studies that were not found by the electronic research. All search results were presented to an expert in the field of Exergames to identify additional literature. The criteria for selecting the expert were: a documented and intensive research in Exergames, successfully acquired third-party funds and projects as well as an extensive list of publications that were thematically located in the research area.

### Data collection and analysis

The reference system CITAVI 6.14 (Wädenswil, Switzerland) was used for literature management and analysis.

#### Selection of studies

After removing duplicates, two reviewers independently screened title and abstract of the identified literature for inclusion. Full texts were then evaluated to determine the studies meeting the selected inclusion criteria. Any disagreement during the selection process was resolved by discussion or by a third reviewer performing a consensus analysis.

#### Data charting process

The data charting of eligible studies was carried out by two reviewers independently using a data extraction sheet (S1 Table). This sheet included:

- key information comprising the developed PICOS criteria (participants, interventions, comparisons, outcomes, study design)
- methodological quality of the identified study (PEDro scale) [38]
- exercise prescription applied in the identified study (frequency, intensity, type, time, volume, and progression–FITT-VP-principles) [6].

Any disagreement during the selection process was resolved by discussion or by a third reviewer performing a consensus analysis. In case of missing data, the original study authors were contacted by e-mail and asked to provide full reports of their methods and results.

#### Assessment of risk-of-bias in the identified studies

Two reviewers independently evaluated the risk-of-bias (RoB) of each included study using an adapted PEDro scale [38]. The assessment of RoB comprised the analysis of 11 criteria including presented eligibility criteria, randomized and concealed allocation of participants, similar baseline, blinding of participants, therapists and assessors, adequate follow-up, intention-to-treat, between group statistical comparisons, and reporting of point and variability measurements [38]. All criteria were judged either as “fulfilled” representing no RoB or as “not fulfilled” representing a potential RoB. To enhance inter-rater reliability, all authors evaluated one of the studies selected in advance. The results of the following discussion formed the basis for the evaluation of all studies selected. Any disagreement was resolved by discussion or by a third author performing consensus analysis.

The overall judgement of the RoB was subsequently performed according to Cashin and McAuley [39]. Thus, the ratings of item 2 to 11 were added to a total score indicating methodological quality as stated:

- Poor: PEDro Score of below 4
- Fair: PEDro Score of 4–5
- Good: PEDro Score of 6–8

Excellent: PEDRo Score of above 8 The mean difference (M) and standard deviation (SD) from tables and text were charted as measure for treatment effects. M and SD from graphs and figures were extracted as treatment effects using the measurement tool provided by Adobe Acrobat Reader (Version 2023.003.20269; Dublin, Republic of Ireland). Only data comparing baseline to post-intervention tests were recorded. In addition, data from follow-up tests were recorded where available.

#### Syntheses of results

The charted data were categorized according to PICOS. The outcomes *knowledge* and *complex motor control/ coordination* were removed from analysis as the literature search did not provide any results, neither for the absolute nor for the relative training effects.

The sample sizes of the included studies were classified using the results of a review published in 2020 [40]. In the studies included in this reference publication, the mean effect size was 0.2 for relative effects (10 studies, 16 outcomes) and 0.7 for absolute effects (17 studies, 32 outcomes). On this basis, optimal sample sizes were calculated using *GPower* (Test: ANOVA: repeated measures, within-between interaction; A priori: computed sample size; α err prob = 0.05; Power (1-β err prob) = 0.75–0.85; Number of groups = 2; Number of measurements = 2; correlation among repeated measurement: 0.5; nonsphericity correction ε = 1). Thus, the optimal total sample sizes were calculated as 46 to 60 participants for the analysis of relative effects and 8 participants for the analysis of absolute effects. The sample size of the included studies was classified as small or large if the number of participants were smaller or larger than the optimal sample size. The *GPower* protocol of the performed calculations can be found in the S2 Text. In studies with more than one control group, the number of participants was adjusted according to the corresponding effects (relative vs. absolute).

Mean differences and 95% confidence intervals were calculated for continuous outcomes according to Fritz, Moris, and Richler [41]. Effect sizes and power were calculated if all relevant data were provided by the authors. Graphical analyses were performed using evidence mapping.

### Differences between protocol and review

The analysis of the results of the systematic literature search revealed that the number of relevant RCT-studies per outcome was low. Furthermore, no clear conclusion could be drawn due to varying results. Thus, a systematic review was deemed to be not suitable. A scoping review including evidence mapping was performed instead. Therefore, neither an analysis of certainty of evidence (GRADE [42]) nor a meta-analysis including subgroup, moderator, or sensitivity analyses were performed. Graphical analyses using forest plots were not performed. Evidence mapping was performed instead. The electronic and manual search was updated on September 4^th^ 2023 to include the latest studies. This scoping review was not registered prior to preparation.

No check of language bias was performed due to lack of studies written in different languages. After screening title and abstract of identified literature, only one study was excluded written in Portuguese. Deviating from the protocol, Science Research was not used in the initial search due to server errors. However, the deep web search engine was included in the updated literature search.

Agility training was categorized as speed training [43]. Analyses of literature revealed that agility tests rather address speed and not flexibility.

## Results

### Description of studies

#### Results of the search

**Fig 1:**
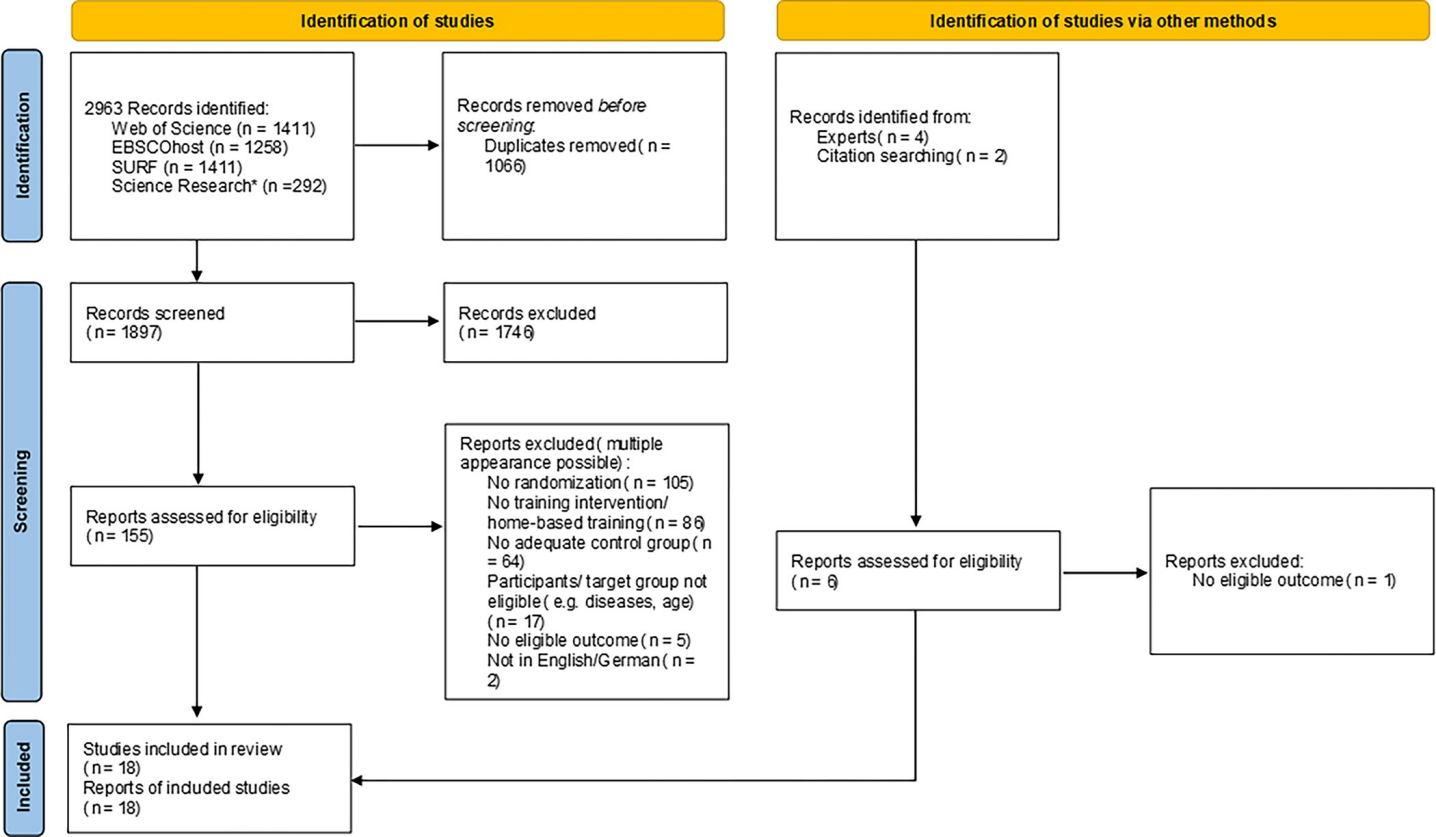
PRISMA flow diagram [43] *Due to server errors during the first search, only results from the second search included.

In total, 2,963 results were identified in the electronic search. The manual search resulted in six additional hits. In total, 1,066 duplicates were removed. After screening abstract and title of the identified literature, another 1,746 results were removed due to various reasons. Full texts of the remaining 155 papers were analyzed resulting in eighteen eligible studies for the review. The screening and selection process of relevant literature is illustrated in the PRISMA flow diagram [44] (see Fig 1).

#### Included studies

In total, eighteen publications met the inclusion criteria [45–62]. A summary of findings of the included studies can be found in Table 2. In total, six studies only analyzed absolute training effects and seven studies only analyzed relative training effects with Exergames. Five studies analyzed the effects of Exergame training compared to a traditional training and to a no treatment control group.

**Table 2:** Summary of findings of included studies.

ID	CG	N	sex	N Judgement	Applied training	Gaming Platform & *Games*	Conventional training	V	Int	Progr	PEDro (see also Table 3)	Main findings
[45]	A	44	f, m	A (RE): small	EG: TRAD: Fitness	XBOX Kinect *Xbox Adventure*	weight shifting, jumping, squatting, boxing, kicking	360			**4**	Compared to TRAD • Mean HR **↔** • static balance **↑↔** • Flow state scale **↑↔** • Social influence, behavioral intention, performance expectancy **↑** • Facilitating conditions, effort expectancy **↔↑**
[46]	P	30	f, m	P (AE): large	EG: Endu-rance	Playpulse Cycling Platform		720			**6**	Compared to CG: • Resting HR **↔** • VO_2_peak/ BW **↑** • VO_2_peak absol. **↔** • SBP **↔** • DBP **↓** • MPA, VPA, MVPA **↔** • VVPA **↑**
[47]	A, P	25	f, m	A (RE): small P (AE): large	EG1, EG2, TRAD: balance	Nintendo Wii *Wii Fit*, *Dance Dance Revolution*	DynaDisc®; DynaDisc® ORBITS.	180	**+**		**4**	Compared to TRAD • dynamic balance **↑↔**
[48]	P	70	f, m	P (AE): large	EG: skill	Motion based dart game	instructions; shooting practice	1440			**5**	Compared to CG: • Shooting score **↑**
[49]	A	22	f, m	A (RE): small	EG, TRAD: balance	Wobble Board therapeutic exergaming system *Neverball*	Wobble Board®	180		**+**	**4**	Compared to TRAD: • Dynamic balance **↔**
[50]	P	22	m	P (AE): large	EG: fitness	XBOX Kinect *Reflex River*, *Dance Central*, *Volleyball*		675			**5**	Compared to CG: • Reaction Time **↑** • Static balance **↔↑** • Dynamic balance **↑**
[51]	A, P	27	?	A (RE): small P (AE): large	EG, TRAD: skill	Nintendo Wii *Tiger Woods PGA Tour*	instructions; golf putts on artificial green	120		**+**	**6**	Compared to CG: • Golf put performance **↑** • Compared to TRAD • Golf put performance **↓**
[52]	P	113	f, m	P (AE): large	EG: fitness	XBOX Kinect *Your Shape*: *Fitness Evolved*	-	1080		**+**	**6**	Compared to CG: • Resting HR **↔** • three-minute step test **↔** • Vital capacity **↔** • SBP, DBP **↔** • Sit-up test **↑** • Back strength, hand grip strength, push-ups, long jump **↔** • Sit and reach **↔** • Response time, side step count **↔** • Static balance **↔** • Dynamic balance **↑↔**
[53]	A	47	f, m	A (RE): optimal	EG, TRAD: fitness	XBOX Kinect *Just Dance*, *Kinect Sports*, *Shape Up*, *Zumba Fitness*	instructor-led exercises in groups; best practices	900			**4**	Compared to TRAD • autonomy, relatedness, competence, exercise enjoyment **↔** • PA level **↔** • Intrinsic motivation, identified regulation, introjected regulation, amotivation **↔**
[54]	A	30	f, m	A (RE): small	EG, TRAD: balance	Nintendo Wii *Wii Fit Plus*	Biodex Balance System	180			**6**	Compared to TRAD: • Static balance **↔**
[55]	A, P	93	f, m	A (RE): large P (AE): large	EG, TRAD: skill	Nintendo Wii *Deca Sports 3*	instructions; supervised games of Racquetball	300			**4**	Compared to CG: • Racquetball skills **↔↑** • PA Level, self-efficacy, future PA intention **↔** Compared to TRAD • Racquetball skills **↔** • Physical enjoyment **↔↑** • PA Level, Self-efficacy, future intention for PA **↔**
[56]	A, P	55	f	A (RE): optimall P (AE): large	EG, TRAD: fitness	XBOX Kinect *Your Shape*: *Fitness Evolved*	Plyometric training (balanced strength and conditioning exercises)	1440		**+**	**4**	Compared to CG: • shuttle run test **↔** • triple hop test **↑↔** • six meter hop test **↔** Compared to TRAD • Shuttle run **↔** • triple hop test, six meter hop test **↔**
[57]	A	32	f, m	A (RE): small	EG; TRAD: strength	BlackBox VR *BlackBox Immersive Virtual Reality*	Cable/ pulley-based crossover machine (dual-selectorized weights)	1260–2160	**+**	**+**	**7**	Compared to TRAD: • SBP, HRV **↑** • DBP, RMR **↔** • VO_2max_, VO_2max_/BW **↑** • 1RM squad, 1RM bench press **↔** • 3RM lat pulldown, 3RM standing row, 3RM squad, 3RM stiff leg deadlift **↑** • 3RM overhead press, 3RM standing chest press **↔** • leg peak power **↑** • Muscle endurance squad **↔** • Muscle endurance bench press **↑** • Sit and reach **↔**
[58]	A, P	90	f, m	A (RE): optimal P (AE): large	EG_SDTon, EG_SDToff: fitness	In-house developed platform *Olympus*	Self-selected training time per session	184–241			**6**	SDTon compared to CG: • MVPA **↑** • Sedentary, light PA **↔** SDToff compared to CG: • MVPA **↑** • Sedentary, light PA **↔**
[59]	P	29	f, m	A (RE): small	EG, TRAD: balance	Nintendo Wii *Wii Fit–balance games*		810		**+**	**5**	Compared to TRAD • Max. isometric power, explosive force leg extensors **↔** • Static balance, dynamic balance **↔**
[60]	A	24	f	A (RE): small	EG: fitness TRAD: balance	XBOX Kinect *Zumba Fitness*	Static & dynamic balance exercises	160			**5**	Compared to TRAD • Static balance **↔** • Dynamic balance **↑**
[61]	P	43	f, m	P (AE): large	EG: fitness	XBOX Kinect *Xbox Adventure*		360		**+**	**4**	Compared to CG: • Side step count **↑** • dynamic balance **↑**
[62]	P	80	f, m	P (AE): large	EG: fitness	Nintendo Switch *Ring-Fit-Adventure*		360			**5**	Compared to CG: • 1600m run **↔** • Heart performance indices **↔**

CG: type of control groups; A: Active control groups/ relative effects (RE); P: passive control group/ absolute effects (AE); N: sample size; V: Total training Volume [min] in the Exergaming and traditional training group; Int: Intensity matched in all active groups; Progr: progression performed; f: female; m: male;?: no information provided; EG: Exergaming group; TRAD: traditional training group; HR: heart rate; VO_2_: oxygen consumption; SBP: systolic blood pressure; DBP: diastolic blood pressure; PA: physical activity; MPA: moderate physical activity; VPA: vigorous physical activity; VVPA: very vigorous physical activity; MVPA: moderate to vigorous physical activity; RMR: Resting Metabolic Rate; HRV: heart rate variability; BW–body weight; SDT: self-determination theory; EG_SDTon: Exergaming with additional SDT features; EG_SDToff: Exergaming without additional SDT features; EG_passive: Gaming with passive inputs.

In total, the studies involved 907 adults. The number of participants per study ranged from 22 participants [49, 50] to 121 participants [58]. All participants were healthy and neither suffered from cardiovascular diseases nor neural or mental disorders. No participant was previously injured.

Mean percentage of males and females in the included studies was not calculated due to missing information provided by the authors.

*Characteristics of included studies*. ***Barry et al*. *(2016)*.** Only active participants performing at least three moderate to vigorous PA sessions per week without experience in playing XBOX were included in th*e* study *by Barry et al*. [45]. The control group training was matched for sequencing, intensity, duration, and progression to the Exergaming group. Training volume was set to three 30 min sessions per week for four weeks.

In this study, baseline differences in postural sway were present. Thus, the Exergaming group showed higher pretest values in the center of pressure displacements. In both exercise groups, moderate to vigorous levels of PA were elicited. The study was performed in the United Kingdom, Europe.

#### *Berg et al*. *(2022)*

In the study performed by Berg et al. [46], only inactive participants performing less than 150 min of moderate PA each week were included. The participants were allocated to the groups by blind card randomization. Participants in the Exergaming group played a variety of Exergames under supervision using the playpulse platform. Training volume was set to twice at least 45 min per week for eight weeks.

PA levels were calculated by wearing activity monitors (SenseWear, Body-Media, Pittsburg, Pennsylvania, United States of America—USA) for consecutive days prior to and after the intervention. However, the days taken into account for calculation varied between the participants from two to seven days indicating a potential bias. Player Experience was excluded from the results as only the Exergaming group was tested and thus no group comparisons were provided. The authors stated that no adverse events occurred during the study. The study was conducted in Norway, Europe.

#### *Brumels et al*. *(2008)*

Only participants that were not engaged in strength or endurance training were included in the study performed by Brumels et al. [47]. No information regarding the PA level of the participants was presented. All three active training groups performed the same type of training that was matched for the time performing balance exercises. Training volume for the participants was set to three 12 to 15 min sessions per week for four weeks.

Unfortunately, the authors presented only selected results regarding balance. Therefore, baseline imbalances cannot be ruled out due to the lack of provided data. The study was conducted in the Netherlands, Europe.

#### *Eliöz et al*. *(2016)*

Eliöz et al. [48] included only participants with experience in shooting training. No further information regarding the PA level or the performance level of the participants was presented. In this study, the control group and intervention group performed the same shooting training. In contrast to the control group, the intervention group additionally performed an Exergame training. Training volume was set to three 30 min sessions per week for 16 weeks.

In both gender groups, baseline differences regarding the shooting scores are present. The study was conducted in Turkey, Asia (Europe).

#### *Fitzgerald et al*. *(2010)*

In the study performed by Fitzgerald et al. [49], training volume was set to 15 min sessions for a total of 12 sessions. Most participants completed the intervention after four weeks performing three sessions per week. However, some participants only completed the training after 5 weeks due to missed sessions. The study was conducted in Ireland, Europe

#### *Hastürk et al*. *(2022)*

Only males who played sedentary video games at least once a week and were not engaged in PA during the study were included in Hastürk et al. [50].

The Exergaming group was further divided in 4 groups with three or two participants per group allowing the participants to play at the same time. Thus, the setting with three available Exergaming stations in one room allowed the participants to play in groups. The three Exergames were played consecutively in the same order for 15 min each. Training volume was set to three 45 min sessions per week for 5 weeks. The study was conducted in Turkey, Asia (Europe).

#### *Heinen et al*. *(2009)*

Only novice participants without golf putting experiences were included in the study by Heinen et al. [51]. No information regarding the PA level or gender of the participants was provided. Both training groups performed the same type of training matched for time training, amount of performed golf putts, and variance of demands while putting (e.g. varying distances and angles of inclination to the hole). Furthermore, both active groups performed the training with a real putting club. Training volume was set to two 20 min sessions per week for three weeks. The study was conducted in Germany, Europe.

#### *Huang et al*. *(2017)*

In the study by Huang et al. [52], Training volume was set to three 30 min sessions per week for 12 weeks. As the type of training was classified as fitness training, a variety of outcomes were presented. This study was performed in Taiwan, Asia.

#### *Hwang et al*. *(2022)*

The study by Hwang et al. [53] only included sedentary participants performing less than 150 min of moderate to vigorous PA per week. The type and time for training was stated to be similar in the Exergaming and the traditional training group. Training volume was set to three 50 min sessions per week for six weeks.

However, the training setting was highly varying in both training groups. The Exergaming group self-regulated their exercise including choosing which Exergame to play, as well as playing alone or with other participants without supervision. In contrast, the traditional training group performed instructor-led exercises in groups. The author also stated that the instructors were advised to exercise with the participants, integrate motivating elements, and provide encouraging feedback. Furthermore, the activities in the traditional group were varying each week or selected to maximize training time. Thus, traditional exercises included dynamic warm-ups, use of gym machines, circuit training, jump-rope workout, step aerobics, high-intensity interval training (HIIT) on bikes, partner-assisted workouts, or cool down with stretching exercises.

As no data regarding the self-reported exertion were provided by the authors, Rate of perceived exertion (RPE) [63] was excluded from further analysis. The study was conducted in the USA, North America.

#### *Ibrahim et al*. *(2015)*

In the study by Ibrahim et al. [54], training volume was set to three 15 min sessions per week for four weeks. The study was performed in Egypt, Africa (Asia).

#### *Jenney et al*. *(2013)*

Only sedentary participants that did not perform more than 20 min of vigorous PA for three days a week or more than 30 min of moderate PA for five days a week were included in the study performed by Jenney et al. [55]. Furthermore, only participants with little knowledge or experience in Racquetball were included. Training volume was set to two 30 min sessions per week for five weeks. As the Exergaming group and the traditional training group performed the same training starting from week five of the intervention, only the results of the first four weeks were included in analysis. The study was conducted in the USA, North America.

#### *Lobato et al*. *(2020)*

Lobato et al. [56] only included moderately trained females performing at least 30 min of exercise per week. No further information regarding the distinction between active and sedentary lifestyle was provided. Traditional plyometric training consisted of balanced strength and conditioning exercises including squat jumps, lungee jumps, jumps over barriers, 180° jumps, or single-leg jumps with holding a specific landing position. Training volume was set to three 60 min sessions per week for eight weeks. The outcome measure used in this study were classified according to Haff and Triplet [33]. In addition to a variety of outcome measures, Lobato et al. [56] used the in-game assessment of the *Nike+ Kinect Training* to determine the fitness and athleticism level of the participants. These tests include assessment of balance, agility, strength, and flexibility. As no data were provided for specific outcomes, these measures were excluded from the results. The study was conducted in Brazil, South America.

#### *Mologne et al*. *(2023)*

Only sedentary participants performing less than four workouts per month were included in the study by Mologne et al. [57]. Training volume was set to three sessions for 35 to 60 min per week for 12 weeks. Exercise choices, exercise sequences, sets, repetitions, and training volume of each participant in the Exergaming group was matched to a control participant to avoid dose-response bias.

PACES [37] data of the participant were excluded from the results, as incomplete data were provided by the authors. The author stated that no adverse events occurred. The study was performed in the USA, North America.

#### *Peng et al*. *(2015)*

The study by Peng et al. [58] only included participants that were playing video games for at least one hour per month and that were not highly physically active. No specific criteria were mentioned for explanation. The two Exergaming-groups played the identical Exergames with varying self-determination-theory features turned on. Thus, the type of training was identical in the active groups. The passive group played the identical game passively with traditional Xbox 360 controller. Training volume was set to three times per week for four weeks. As the participants were able to self-determine their training time, the total time was varying between the groups. As no data was provided for the PACES [37], this measure was excluded from the results.

The authors state that the data collection was conducted in two waves. Thus, the authors discuss a seasonal effect of the weather on moderate to vigorous PA. The study was conducted in the USA, North America.

#### *Röttger et al*. *(2011)*

Röttger et al. [59] included only untrained or inactive participants were in their study. No information regarding the PA level of the participants was provided. Training volume was set to three times 45 min per week for 6 weeks. The study was conducted in Germany, Europe.

#### *Shahvali et al*. *(2021)*

The study performed by Shahvali et al. [60] only included sedentary females with a score below 600 in the international physical activity questionnaire [64]. The type of training was varying in the Exergaming and the active control group. In this study, a conventional balance training was performed in the active control group. According to the authors, the use of Zumba software in the Exergaming group was also considered as balance training in this scoping review. However, this type of training was classified as fitness training. Training volume was set to two 20 min sessions per week for four weeks. The study was conducted in Iran, Asia.

#### *Su et al*. *(2015)*

In the study by Su et al. [61], training volume was set to three sessions per week with an average of 20 min per session for four weeks. Only selected results regarding balance were presented by the authors. The study was conducted in Taiwan, Asia.

#### *Wu et al*. *(2022)*

Training volume was set to three 30 min training sessions per week for four weeks in the study performed by Wu et al. [62]. The study was performed in Taiwan, Asia.

#### Studies awaiting assessment/ ongoing studies

No studies awaiting assessment or ongoing studies were found.

#### Excluded studies

In the first selection process (screening title and abstract), the main reasons for exclusion were the presence of diseases, disorders or injuries, participants’ age below 18 or above 64, or irrelevant literature (e.g., technology developments).

In the full text screening, the main reasons for exclusion were:

- no training intervention
- the lack of an appropriate control group (e. g. application of a repeated measures or cross-over study design)
- the lack of randomization of the participants

Excluded studies and their reasons for exclusion can be found in the “Characteristics of excluded studies” in the S2 Table.

### Risk of bias in included studies

Overall, a potential risk-of-bias (RoB) was found in all included studies. Thus, the methodological quality of the studies was varying between fair and good (scores: 4 to 7).

In total, twelve studies were rated as fair [45, 47–50, 53, 55, 56, 59–62]. Six studies were rated as good [46, 51, 52, 54, 57, 58]. The full judgement of RoB in included studies is displayed in Table 3.

**Table 3:** Risk of bias [3,8] in included studies–remark: High values indicate low risk; Item 1 was not included in the total score.

Item	1	2	3	4	5	6	7	8	9	10	11	
reference	eligibility criteria	random allocation participants	allocation concealed	similiar baseline	blinding of subjects	blinding of therapists	blinding of assessors	> 85% of subjects completed	"intention to treat"	between group statistics	point & variability measurements	SCORE
[45]	x	x						x		x	x	**4**
[46]	x	x		x				x	x	x	x	**6**
[47]	x	x						x		x	x	**4**
[48]	x	x						x	x	x	x	**5**
[49]	x	x		x						x	x	**4**
[50]	x	x						x	x	x	x	**5**
[51]	x	x		x				x	x	x	x	**6**
[52]	x	x			x		x	x		x	x	**6**
[53]	x	x	x							x	x	**4**
[54]	x	x		x				x	x	x	x	**6**
[55]	x	x						x		x	x	**4**
[56]	x	x		x				x		x		**4**
[57]	x	x	x	x				x	x	x	x	**7**
[58]	x	x	x	x			x			x	x	**6**
[59]	x	x		x				x		x	x	**5**
[60]	x	x						x	x	x	x	**5**
[61]	x	x		x						x	x	**4**
[62]	x	x		x				x		x	x	**5**
N of studies	18	18	3	10	1	0	2	14	7	18	17	
% of studies	100	100	16.7	55.6	5.5	0.0	11.1	77.8	38.9	100	94.4	

Although all included studies were randomized, controlled trials, most identified studies did not specify the method of randomization. One study performed blind card randomization [46]. Two studies used computer generated random numbers [57, 58]. Two studies randomly allocated participants to the groups after stratifying for sex [46, 55]. All studies explicitly presented the eligibility criteria for participants except for Brumels et al. [47].

Two studies explicitly reported the use of the “intention-to-treat” principle [46, 61]. However, Su et al. [61] stated that missing data were imputed by transferring the last recorded observations. As no further information was provided, these data were classified as “no intention-to-treat”. Furthermore, seven studies did not report any dropouts throughout the study [48, 51]. Thus, these studies were judged to apply the intention-to-treat principle.

### Effects of interventions

All findings were categorized according to the corresponding outcome.

A list of identified measures used in the studies categorized per outcome and their corresponding reliability is documented in the S3 Table. No reliability check was performed for the measuring devices used to record the outcomes as reliable instruments were assumed.

Effect sizes d and confidence intervals were calculated for all measures if available. As not all authors provided relevant data, the findings will only be included in the evidence maps. Calculated effect sizes d and calculated confidence intervals for all measures categorized per outcome and authors can be found in the S3 Text. In Table 4, mean effect sizes, range of calculated effect sizes and number of included measures for each outcome are presented.

**Table 4:** Mean effect sizes and range of effect sizes for all measures categorized according to the corresponding outcome.

Outcome		Absolute effects (passive control group without intervention)			Relative effects (active control group with traditional training intervention)		
		d	Range (Min–Max)	# of summarized measures	d	Range	# of summarized measures
Primary	endurance	-0.01	-0.74–0.25	15	0.38	-0.16–0.85	7
	strength	-0.03	-0.26–0.48	5	0.55	-0.03–1.39	13
	flexibility	0.10	0.10–0.10	1	0.40	--	1
	Speed	0.21	0.01–0.55	4	No data		
	Balance	0.55	-0.18–1.28	9	0.40	0.15–1.44	15
	Skills	2.10	1.95–2.25	2	-0.79	--	1
	Player Experience	No data		0	0.26	-0.15–1.33	15
Secondary	Adverse events	No adverse events occurred			No data		
	PA level	0.37	-0.24–0.85	11	0.03	-0.07–0.11	3
	Attitudes	1.19	1.19–1.19	1	0.36	-0.14–0.95	10

#### Evidence mapping

**Fig 2:**
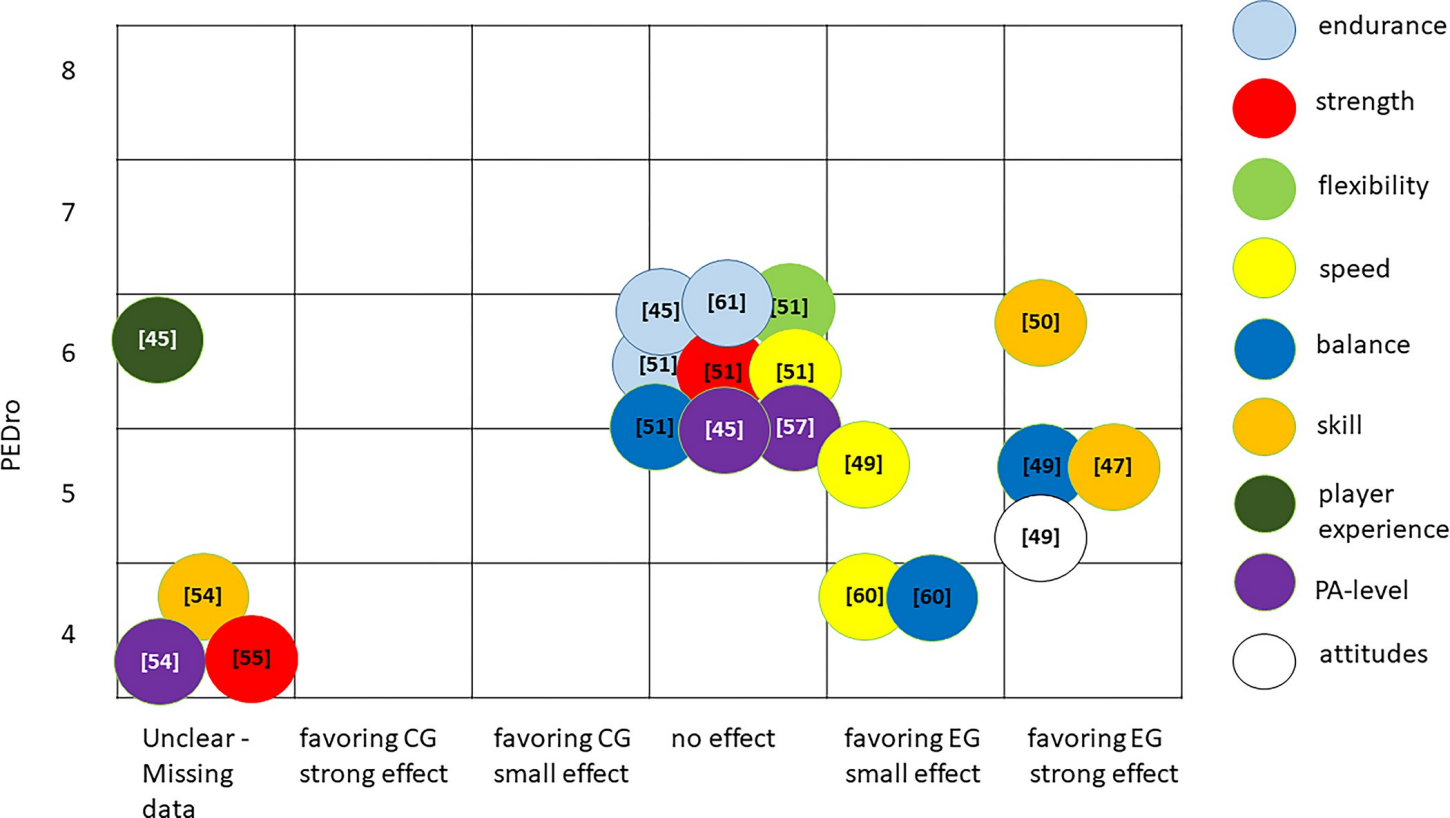
Evidence map synthesizing the strength of evidence regarding absolute effects of primary and secondary outcomes included in analysis. y-axis; risk-of- bias of studies (adapted PEDro-scale), x-axis: summarized estimated effect of particular outcome; size of bubble: judged sample sizes.

**Fig 3:**
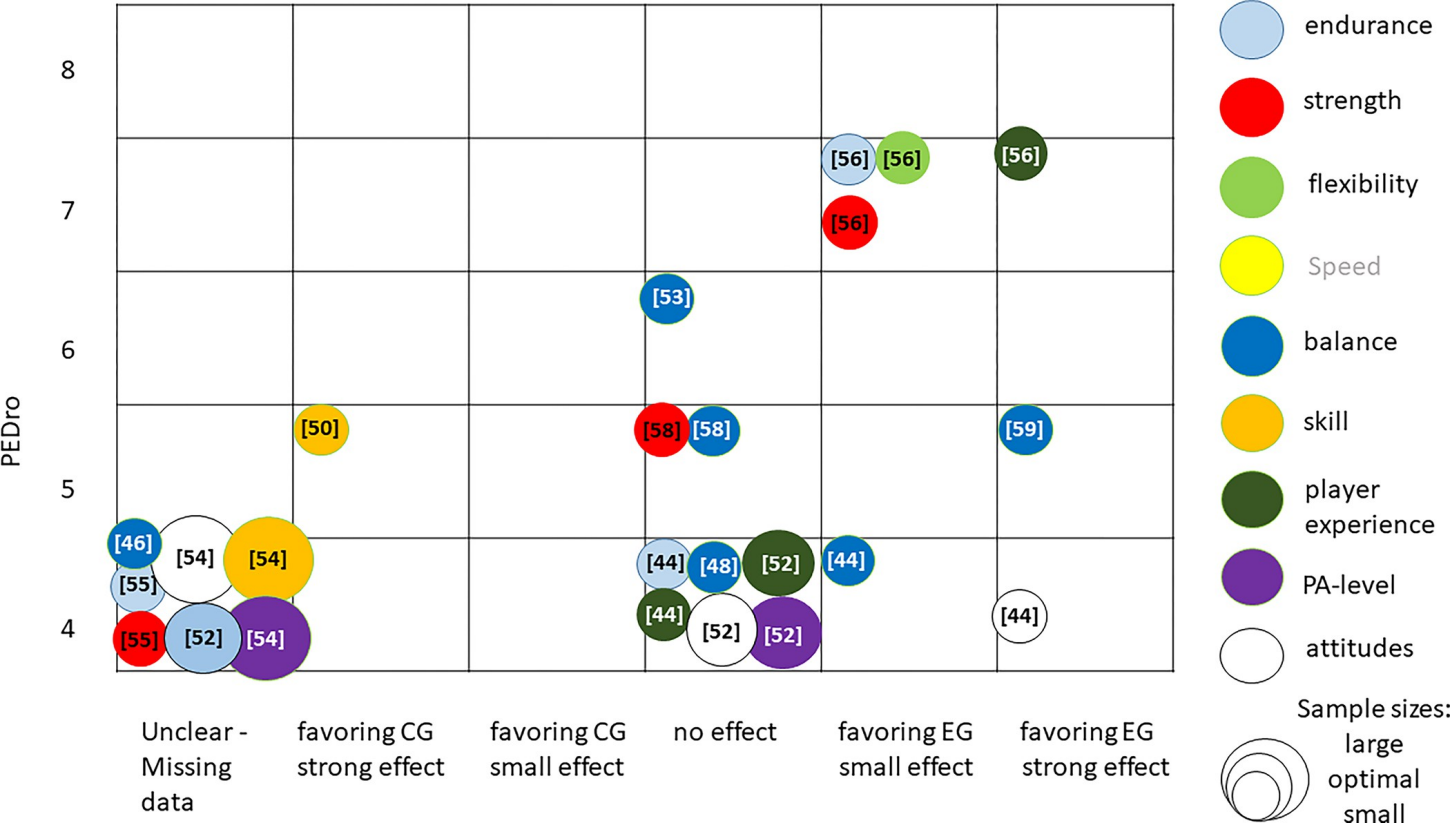
Evidence map synthesizing the of strength of evidence regarding relative effects of primary and secondary outcomes included in analysis. y-axis; risk-of- bias of studies (adapted PEDro-scale), x-axis: summarized estimated effect of particular outcome; size of bubble: judged sample sizes.

Figs 2 and 3 provide visual representation of the summarized absolute and relative training effects of primary and secondary outcomes of the included studies categorized according to the corresponding outcome. The bubble blots depict the estimated research volume based on the estimated RoB (y-axis; adapted PEDro-scale [38]), the estimated effect (x-axis), and the classified sample sizes. As all three dimensions are estimates, only a broad overview of the evidence base is provided. Outcomes that were analyzed but insufficiently reported by the authors were integrated in the evidence maps as missing data.

Regarding absolute training effects, the effect sizes of included studies varied from no effect to large effects favoring Exergames. No study was found with training effects favoring the no training control group. All sample sizes of included studies were judged as large. Only strong effects favoring Exergame training were found for the outcomes skill and attitudes. The summarized effect sizes of strength, endurance, and flexibility training with Exergames revealed no effect compared to no training. Fig 2 shows a large research gap in high quality studies analyzing the absolute effects of Exergame training. Furthermore, absolute effects of Exergame training on flexibility, strength, player experience, and attitudes are insufficiently investigated.

Relative training effects of Exergame training compared to traditional training displayed in Fig 3 show a similar picture. In general, the relative training effects varied from no effect to strong effects favoring Exergaming. Only one exception occurred. One study found strong effects favoring the traditional training group in skill training. As only one study with a PEDro score of seven was found, a research gap with high-quality studies can also be seen in the relative effects. The highest number of studies were found for the relative effects of Exergame training on balance and player experience. Effects of flexibility training with Exergames was only investigated in two studies. Furthermore, no study was found analyzing the relative effects of Exergame training on speed or agility.

## Discussion

The aim of this scoping review was to identify available evidence for absolute and relative effects of training with Exergames on physical indicators (effectiveness) and motivation (attractiveness) compared to no or conventional training in healthy adults and to identify gaps in the current evidence.

The systematic literature search revealed only eighteen studies meeting the defined eligibility criteria. Furthermore, the number of studies per outcome was varying between only two studies (e.g. one per absolute effects: flexibility; one per relative effects: flexibility) and six studies (relative effects: balance). Furthermore, no study was found analyzing the relative effects on speed. Thus, the current state of research regarding the absolute and relative effects of Exergames in healthy adults is still limited although the total number of studies regarding Exergames increased in the last decades. This becomes particularly evident as not all primary outcomes have yet been addressed in randomized controlled trials. Therefore, not only a lack of reviews addressing the broad effects of training with Exergames or AVG in healthy adults is present but also the lack of high quality and comparable studies in general. The summarized, absolute effects of Exergame training ranged from no effect to large effects for primary and secondary outcomes. Especially in studies analyzing the effect of exergaming on specific athletic skills, significant non-zero and thus substantial effect sizes were found. This effect was independent of the performance level of the participants and the applied parameter for training control. In particular, Eliöz et al. [48] found a high absolute effect on shooting skills in experienced shooters training 30 min three times each week for a total of 16 weeks. Heinen et al. [51] found high absolute effects of Exergame training on putting performance in novice golfers. In contrast to the study by Eliöz et al. [48], the participants only trained twice a week for 20 minutes over three weeks. However, Heinen et al. [51] applied a systematic variation of demands to improve the training efficacy. In another study [55], Exergame training was performed twice a week for 30 min over 5 weeks. Although no effect size was calculated due to missing data, an effect of Exergame training on Raquetball Skill Test Performance [65], was found. As no effect was found in the control group, a positive absolute effect favoring Exergame training can be assumed.

Furthermore, non-significant, high and moderate effects were found for selected outcome measures (VO_2_ peak relative to body weight [46], or sit-up test [52]). Although positive changes were found for further parameters, the effect sizes for these parameters were small or inconsistent. These inconsistent results can be observed particularly in studies analyzing the absolute effects of Exergame training on balance and speed. Hastürk et al. [50] and Su et al. [61] consistently found moderate, non-significant effects on static and dynamic balance. In contrast, Huang et al. [52] found an effect size of ES = -0.18 on closed eye foot balance. Similarly to the results regarding balance, these varying results can also be observed in the findings regarding speed. Thus, Hastürk et al. [50] and Su et al. [61] found moderate, non-significant effects on reaction time (ES = 0.46) and the side step test (ES = 0.55 [33]), respectively. In contrast, the study by Huang et al. [52] found a negative effect size in response time (ES = -0.19) and no effect on the side step test (ES = 0.01 [33]). All three studies used the XBOX Kinect as training device with varying Exergames. The male participants in the study by Hastürk et al. [50] performed an Exergame training three times a week for 45 min over 5 weeks. Su et al. [61] also applied an Exergame training for only 20 minutes three times a week over 6 weeks. In contrast, the training volume in the study by Huang et al. [52] was the highest with training three times a week for 20 min over twelve weeks. However, the calculated effect size might be caused by the high improvements comparing pre- and post-test in both the Exergaming and in the control group. Therefore, a habituation effect to the testing procedures might be possible biasing the results. Additionally, further negative effect sizes favoring the control group were found for selected outcome measures. In particular, Berg et al. [46] found negative effects for systolic and diastolic blood pressure, as well as for moderate and moderate to vigorous PA. However, the negative effect on blood pressure might be caused by a higher difference between pre- and post-test in the control group compared to the exergaming group. In contrast to the control group, the values in the exergaming group remained rather constant. Furthermore, no significant differences were found in both groups in the post-test indicating possible baseline differences. Additionally, in contrast to the negative effects on moderate and moderate to vigorous PA, a significantly high effect was found for very-vigorous PA. Thus, the time spent with PA shifted to more intense PA. In contrast, the amount of time spent with PA was constantly decreasing in the control group independent of intensity.

Only the study by Hastürk et al. [50] analyzed the absolute effects of Exergaming on attitudes. Similar to the results presented regarding speed and balance, strong effects favoring the Exergaming group on mental well-being [66] and subjective happiness [67] were found. However, the sample size consisted exclusively of male participants. Therefore, the findings need to be confirmed in further studies integrating more differentiated samples.

The summarized, relative effects of Exergame training compared to traditional training also ranged from no effect to large, with one exception. In contrast to the absolute effect favoring Exergaming, a strong effect favoring the traditional training group was found for skill training in Heinen et al. [51]. Furthermore, inconsistent results were found for endurance, strength and balance.

For the relative effects on endurance, consistently moderate effects favoring Exergaming were only found by Mologne et al. [57], with one exception (diastolic blood pressure). In contrast, the studies by Barry et al. [45] and Lobato et al. [56] did not find an effect on mean HR or the shuttle run score. However, the training characteristics were highly varying in the corresponding studies. First, Mologne et al. [57] applied a resistance training instead of a fitness training in the active training groups. Second, Mologne et al. [57] included only inactive participants, whereas the other studies included active participants. Third, the total duration of the intervention was set to 12 weeks instead of four weeks [45] and eight weeks [56].

Methodological differences in the studies by Mologne et al. [57] and Röttger et al. [59] are also reflected in the results for the relative effects of Exergame training on strength. The study by Mologne et al. [57] found predominantly weak to moderate effects favoring Exergaming. In contrast, no effects on force development and the maximum leg force was found in Röttger et al. [59]. However, the participants in the study by Röttger et al. [59] did not perform a specific resistance but a balance training over only 6 weeks.

This lack of specificity in the type of training is also noticeable in the relative effects of Exergame training on flexibility. In particular, Mologne et al. [57] found weak effects on the sit and reach test (ES = 0.40 [33]) while applying a strength training in their study. In total, the improvements in the Exergaming and in the traditional training groups were small since no specific flexibility training was applied. However, the cable-based resistance Exergaming machine used in the Exergaming group adjusted the intensity according to user`s force generation, range of motion and speed of execution. This individualized adjustment was missing in the traditional training group.

The training effect of Exergaming on balance compared to traditional training was the most investigated outcome in the included studies. However, the results were also varying and inconsistent. Significantly different from zero and therefore substantial effect sizes were only found for the Timed Up and Go test [68] by Shahvali et al. [60] representing dynamic balance in this particular study. However, only sedentary females were included. In addition, the study had the lowest total volume of all identified studies on relative effects on balance with 2 times 20 minutes of training per week over 4 weeks. In contrast to the expectations, large and moderate effects favoring Exergame training were only identified in studies that did not perform a specific balance but a fitness training in the Exergaming groups. Barry et al. [45] used the Exergame *XBOX Adventures* (XBOX Kinect) and Shahvali et al. [60] used *Zumba Fitness* (XBOX Kinect). However, in contrast to the participants performing the same type of fitness training in both training groups in Barry et al. [45], the participants in the study by Shahvali et al. [60] performed a traditional balance training in the traditional training group. Brumels et al. [47], Fitzgerald et al. [49], and Ibrahim et al. [54] did not find an effect on balance. All three studies applied the same training volume with three times 15 minutes trainings sessions over 4 weeks. However, the results presented by Brumels et al. [47] suggest an improvement of both Exergaming groups (Wii Fit and Dance, Dance Revolution respectively) over the traditional training group. Due to missing data, a calculation of effect sizes was not possible. The remaining results of the included studies suggest that Exergaming produces similar training effects compared to traditional training. The motivational effects evoked by Exergaming ranged from no to large effects. In general, substantial lower rates of perceived exertion were found in the Exergaming groups [45, 57]. This is in line with previous research and underlines the substantial short-term effect of Exergames on motivation [64–66]. This effect might vanish in the long-term training due to the novelty effect. However, the study by Mologne et al. [57] found a substantial large effect on RPE […], although this study applied the longest intervention duration with three times training per week over 12 weeks.

In contrast, the studies by Barry et al. [45] and Hwang et al. [53] found no effects on Player Experience. Barry et al. [45] used XBOX Adventures for the Exergame training with active participants for three times 30 minutes Exergame training for 4 weeks. Although higher values were found in the Exergaming group, no subscale of the Flow State Scale [69] found an effect favoring Exergames except for Action Awareness Merging (ES = 0.44). In line with the findings of Barry et al. [45], Hwang et al. [53] found no effects on Player Experience in terms of exercise enjoyment and support for the needs of autonomy, competence, and relatedness [70]. Furthermore, the authors presented a continuous increase in exercise motivation in the traditional training group compared to the Exergaming group. However, the authors additionally state a higher increase in autonomy and intrinsic motivation in the Exergaming group. Correspondingly, extrinsic regulation was decreasing in the Exergaming and increasing in the traditional training group. Thus, varying behavioral regulations might influence the long-term effects of exercise enjoyment in Exergame training compared to traditional training. Furthermore, this finding has to be considered with caution as the setting and organization of the training was highly varying in both training groups.

Unfortunately, this varying organization of Exergaming training compared to the traditional training was present in most studies. Thus, the participants assigned to the Exergaming group were mostly training alone. In contrast, the traditional training was often performed in groups led by supervisors. These supervisors were exercising together with the participants, were advised to integrate motivating elements, or provided encouraging feedback. Furthermore, the exercises performed were either varying each week or selected to maximize training time [53]. Another weakness was the inconsistent differentiation between active and sedentary lifestyles in the included studies. Thus, participants in the study performed by Berg et al. [46] were classified as “inactive” when performing less than 150 min of PA per week or not regularly performing endurance training. In contrast, Peng et al. [58] labeled their participants as inactive when they performed less than 225 min of moderate PA per week. Due to these additional influencing factors, the motivational effects caused by Exergames are hardly comparable.

Summarizing the evidence for absolute and relative effects of Exergaming, the overall results are varying and inconsistent in the identified studies. In addition to the already discussed variances of the performed training with different FITT-VP-principles for exercise prescription applied, another reason for these heterogeneous results could be the inconsistent outcome measures in the included studies. On the one hand, this comprises different measures with varying reliability and validity for individual outcomes. On the other hand, identical measures are used to assess varying outcomes. Furthermore, the type of training applied in the Exergames and outcome measures assessed by the authors are occasionally not identical [60]. In addition, the reliability and validity of the included outcome measures were varying.

Furthermore, only few studies used Exergames that provided a specific type of training. Most studies analyzed training effects of non-specific fitness Exergames. As these games combine a variety of components, a precise classification is hardly possible. Additionally, some studies used Exergames that integrate a variety of mini-games without specification regarding the choice of mini-games. For example, Su et al. [61] assessed the effects on balance and agility evoked by the fitness Exergame Xbox Adventures. As the software consists of several individual games and the participants were not given any guidelines regarding the choice of games, the type of training might have varied in individual participants. In addition, the mini-games usually only run for a few minutes before they can be restarted or switched to another game. Therefore, the exact duration of the training with Exergames may not be recorded correctly and thus differ from the specifications. Only the study performed by Mologne et al. [57] matched the choice and sequence of exercises, sets, repetitions, and time-to-complete of participants in the Exergaming group to participants in the traditional training group to prevent dose-response bias. Therefore, the positive relative effects of Exergames training on endurance, strength, and player experience can be considered as valid evidence.

Furthermore, the FITT-VP principles of exercise prescription provided by the ACSM [5, 6] were occasionally neglected in some studies. For example, the only study applying specific endurance training in the Exergaming group had a total duration of only 4 weeks [46]. As morphological adaptations to endurance training can only be reliably observed after 12 weeks of training [71], the duration of the study might be too short to evoke substantial physiological adaptation to training.

Although no moderator analysis was performed, the findings of the authors regarding the moderating role of the baseline PA level will be addressed. In contrast to previous literature [23, 72], the included studies found mixed results for participants with high baseline PA. Particularly in Huang et al. [52], higher effects of Exergaming on selected parameters of endurance, strength, flexibility, and speed were found in participants exercising more than 120 min per week. This also holds for the effects of skill training [55]. These effects could be related to a higher baseline motivation to be active and be familiar with PA. Furthermore, the results confirm the short-term motivational influence of exergaming on individuals with low baseline PA. Thus, the exercises during the intervention consistently increased the duration and intensity of the PA levels in sedentary participants. Due to the lack of follow-up tests, however, no conclusion can be given about the moderating role of PA in the long-term training. Due to these varying results, a general conclusion on the moderating role of PA cannot be drawn.

The addressed methodological weaknesses of the included studies could bias the results of this scoping review. In general, all included studies had a potential RoB in at least one criterion of the PEDro Scale. A further potential bias in the review process was the missing assessment of grey literature. It is possible that further interventions were performed as part of theses, dissertations or specific projects reports. Thus, no ongoing studies or studies awaiting assessment were included in this review. In addition, not all information was provided by the authors resulting in the exclusion of potentially relevant literature.

### Implications for research and practice

For future research, it is essential to extend the knowledge of training with Exergames analyzing the addressed outcomes. This includes not only conducting further RCT studies to analyze the specific effects of Exergame training on endurance, strength, or balance. Outcomes that have been neglected in previous studies, such as sensori-motor coordination, or agility need to be evaluated with caution. In particular, training of sensorimotor coordination as well as executive and motor-functions performed with Exergames has gained increased attention [73]. However, no RCT study was yet found in the literature search.

Furthermore, future research should focus on a high methodological quality in conducting and reporting of studies. Particularly, methodological weaknesses, varying and incomplete reporting of the results were found in all publications. One possibility to improve this is to register and publish a protocol of the planned study or review. Detailed protocols ensure a systematic and well-documented planning of the study, reduce arbitrariness in decision-making, and improve the research integrity and transparency [74].

Furthermore, appropriate guidelines should be used for conducting and reporting (i.e., [26, 29, 30]). This ensures that a minimum list of relevant elements is addressed in a structured way. Due to the double mission of Exergames combining attractiveness and effectiveness, a specific guideline may be required that also includes quality aspects of the used Exergame [15]. Furthermore, only four of the identified studies analyzed both the effectiveness and the attractiveness of Exergames [45, 50, 53, 57]. Thus, future research should pay more attention on the relationship between those aspects. Another important aspect is to standardize the outcome measures per outcome regarding physical and motivational effects in Exergame training. This scoping review provides an initial step for the development of a specified guideline to support future studies.

This review also revealed the substantial lack of specific Exergames to be used as effective and efficient training tools. Currently, only specific balance training can be performed with commercially available Exergames. All other Exergames used in the included studies either combined several training components as fitness training or were not freely released prototypes developed in research projects. Although these games can evoke distinct training effects, the further development of Exergames should pay attention to distinct quality criteria [15, 16].

## Supporting information

S1 Protocol Review protocol training effects Exergames. (PDF)

S1 Text Included databases in literature search. (DOCX)

S2 Text GPower sample size low to moderate-effects. (RTF)

S3 Text Effect sizes and confidence intervals calculated for all measures. (DOCX)

S4 Text Prisma-ScR checklist. (DOCX)

S1 Table Data extraction sheet. (XLSX)

S2 Table List of excluded studies. (XLSX)

S3 Table Outcome measures. (XLSX)
