# Supplementary material for: Physical and motivational effects of Exergames in healthy adults—A scoping review
Source: PLoS One. 2025 Feb 7;20(2):e0312287. doi: 10.1371/journal.pone.0312287 (PMC11805416; doi:10.1371/journal.pone.0312287)
Supplement: S1 Text — (DOCX) [file pone.0312287.s002.docx]

Included data bases of literature search „Physical and motivational effects of Exergames in healthy adults — a scoping review” by K. Hoffmann, J. Wiemeyer & A. L. Martin-Niedecken

**EBSOhost Platform:**

- INSPEC
- Library
- Information Sciences & Technology Abstracts
- APA PsycArticles
- APAPsycInfo
- SPORTDiscus with Full Text
- MEDLINE

**Science Research:**

Computer & Technology:

- AIAA Electronic Library
- Association for Computing Machinery
- Biotechnology Industry Organization
- Institute of Electrical & Electronics Engineers
- National Institute of Standards and Technology Data Gateway
- National Science Foundation
- National Technical Information Service
- NIH RePORTER
- Oxford University Press
- Science Magazine
- Springer
- University of Southhampton, Electronics and Computer Science

Health and Medicine:

- American Association for Cancer Research
- American College of Physicians
- Bandolier
- BioMed Central
- Cell Press
- Center for Disease Control
- Chicago Press - Life Sciences
- ClinicalTrials.gov
- Environmental Health Perspectives
- Eunice Kennedy Shriver National Institute of Child Health and Human Development
- Food and Drug Administration Site
- Journal Watch
- Karger Publications
- Karolinska Institutet
- Mayo Clinic
- Mednar
- National Institutes of Health
- NIH MedlinePlus
- PubChem
- PubMed
- PubMed Central
- Science Magazine
- Springer
- The Journal of the American Medical Association
- U.S. Department of Labor: Occupational Safety & Health Administration
- U.S. Food and Drug Administration
- WebMD
- World Health Organization

Multidisciplinary Sources:

- Annual Reviews
- British Library Direct
- Congressional Research Service
- National Research Council of Canada
- National Science Foundation
- National Technical Information Service
- OAIster
- OpenDOAR
- Oxford University Press
- Scholarpedia
- Science Magazine
- Springer
- Taylor and Francis Group
- Wiley InterScience
