# Supplementary material for: Physical and motivational effects of Exergames in healthy adults—A scoping review
Source: PLoS One. 2025 Feb 7;20(2):e0312287. doi: 10.1371/journal.pone.0312287 (PMC11805416; doi:10.1371/journal.pone.0312287)
Supplement: S2 Text — (RTF) [file pone.0312287.s003.rtf]

[1] -- Thursday, November 09, 2023 -- 11:21:41
F tests - ANOVA: Repeated measures, within factors
Analysis:	A priori: Compute required sample size 
Input:	Effect size f	=	0.2
	á err prob	=	0.05
	Power (1-â err prob)	=	0.75
	Number of groups	=	2
	Number of measurements	=	2
	Corr among rep measures	=	0.5
	Nonsphericity correction å	=	1
Output:	Noncentrality parameter ë	=	7.3600000
	Critical F	=	4.0617065
	Numerator df	=	1.0000000
	Denominator df	=	44.0000000
	Total sample size	=	46
	Actual power	=	0.7559537

[2] -- Thursday, November 09, 2023 -- 11:22:15
F tests - ANOVA: Repeated measures, within factors
Analysis:	A priori: Compute required sample size 
Input:	Effect size f	=	0.2
	á err prob	=	0.05
	Power (1-â err prob)	=	0.85
	Number of groups	=	2
	Number of measurements	=	2
	Corr among rep measures	=	0.5
	Nonsphericity correction å	=	1
Output:	Noncentrality parameter ë	=	9.6000000
	Critical F	=	4.0068729
	Numerator df	=	1.0000000
	Denominator df	=	58.0000000
	Total sample size	=	60
	Actual power	=	0.8614225

[3] -- Thursday, November 09, 2023 -- 11:22:40
F tests - ANOVA: Repeated measures, within factors
Analysis:	A priori: Compute required sample size 
Input:	Effect size f	=	0.7
	á err prob	=	0.05
	Power (1-â err prob)	=	0.75
	Number of groups	=	2
	Number of measurements	=	2
	Corr among rep measures	=	0.5
	Nonsphericity correction å	=	1
Output:	Noncentrality parameter ë	=	15.6800000
	Critical F	=	5.9873776
	Numerator df	=	1.0000000
	Denominator df	=	6.0000000
	Total sample size	=	8
	Actual power	=	0.9064852

[4] -- Thursday, November 09, 2023 -- 11:22:55
F tests - ANOVA: Repeated measures, within factors
Analysis:	A priori: Compute required sample size 
Input:	Effect size f	=	0.7
	á err prob	=	0.05
	Power (1-â err prob)	=	0.85
	Number of groups	=	2
	Number of measurements	=	2
	Corr among rep measures	=	0.5
	Nonsphericity correction å	=	1
Output:	Noncentrality parameter ë	=	15.6800000
	Critical F	=	5.9873776
	Numerator df	=	1.0000000
	Denominator df	=	6.0000000
	Total sample size	=	8
	Actual power	=	0.9064852
