# Supplementary material for: Physical and motivational effects of Exergames in healthy adults—A scoping review
Source: PLoS One. 2025 Feb 7;20(2):e0312287. doi: 10.1371/journal.pone.0312287 (PMC11805416; doi:10.1371/journal.pone.0312287)
Supplement: S3 Text — (DOCX) [file pone.0312287.s004.docx]

**Calculated effect size and calculated confidence intervals for**

**all measures categorized according to the corresponding outcome**

Table. **Calculated effect size and calculated confidence intervals for all measures categorized according to the corresponding outcome**. Legend: * significant effects, HR = heart rate; BW = body weight; MPA = moderate physical activity level; MVPA = moderate to vigorous physical activity; VPA = vigorous physical activity; VVPA = very vigorous physical activity; MVPA = moderate to vigorous physical activity; SDT on = Exergaming with all available features; SDT off = Exergaming with reduced features; SDT_pass = passive gameplay; COP = Center of pressure; ML = medial-lateral; AP = anterior-posterior

| Outcome |  | Absolute effects (passive control group without intervention) | | Relative effects (active control group with traditional training intervention) | | |  |
| --- | --- | --- | --- | --- | --- | --- | --- |
|  |  | d | 95% CI | d | | 95% CI | |
| Endurance | resting HR | 0.21  0.24 | -0.54 to 0.95 [45]  -0.13 to 0.61 [51] |  | |  | |
|  | Mean HR |  |  | -0.07 | | -0.66 to 0.52 [56] | |
|  | Heart rate variability |  |  | 0.85 | | 0.122 to 1.57 [56] * | |
|  | VO_2_ peak | 0.08 | -0.66 to 0.82 [45] | 0.64  0.55 | | -0.07 to 1.36 [56]  -0.15 to 1.27 [56] | |
|  | vital capacity | 0.06 | -0.43 to 0.31 [51] |  | |  | |
|  | Resting metabolic rate |  |  | 0.22 | | -0.47 to 0.92 [56] | |
|  | Systolic blood pressure | -0.74  -0.03 | -1.51 to 0.03 [45]  -0.41 to 0.34 [51] | 0.64 | | -0.07 to 1.35 [56] | |
|  | Diastolic blood pressure | -0.25  -0.29 | 0.98 to 0.47 [45]  -1.51 to 0.03 [51] | -0.16 | | -0.857 to 0.53 [56] | |
|  | Three minute step test | 0.25 | -0.12 to 0.62 [51] * |  | |  | |
|  | 1600m run | 0.24 | -0.15 to 0.73 [61] |  | |  | |
|  | CFIPA walk | -0.27 | -0.71 to 0.17 [61] |  | |  | |
|  | CMPB_walk | -0.27 | -0.71 to 0.17 [61] |  | |  | |
|  | CFIPA_run | 0.11 | -0.33 to 0.55 [61] |  | |  | |
|  | CMPB_run | 0.11 | -0.33 to 0.55 [61] |  | |  | |
| strength | sit-up test | 0.48 | 0.11 to 0.86 [51] * |  | |  | |
|  | back strength | -0.23 | -0.60 to 0.14 [51] |  | |  | |
|  | hand grip strength | -0.20 | -0.56 to 0.18 [51] |  | |  | |
|  | push-ups | 0.02 | -0.35 to 0.39 [51] |  | |  | |
|  | long jump | -0.26 | -0.63 to 0.12 [51] |  | |  | |
|  | Max. isometric power |  |  | -0.03 | | -0.76 to 0.70) [58] | |
|  | explosive force leg extensors |  |  | -0.03 | | -0.76 to 0.70) [58] | |
|  | 1RM chest press |  |  | 0.24 | | -0.44 to 0.95 [56] | |
|  | 1RM squat press |  |  | 0.38 | | -0.36 to 1.04 [56] | |
|  | 85% 1RM chest press |  |  | 1.04 | | 0.30 to 1.78 [56] * | |
|  | 85% 1RM squat press |  |  | 0.32 | | -0.38 to 1.12 [56] | |
|  | Leg Power (Peak - W) |  |  | 1.39 | | 0.61 to 2.16 [56] * | |
|  | 3RM Lat Pulldown |  |  | 0.59 | | -0.12 to 1.30 [56] | |
|  | 3RM Standing Row |  |  | 0.79 | | 0.07 to 1.52 [56] | |
|  | 3RM Overhead press |  |  | 0.48 | | -0.22 to 1.18 [56] | |
|  | 3RM standing chest press |  |  | 0.43 | | -0.27 to 1.13 [56] | |
|  | 3RM squat |  |  | 0.79 | | 0.07 to 1.51 [56] | |
|  | 3RM stiff leg deadlift |  |  | 0.75 | | 0.03 to 1.47 [56] | |
| flexibility | sit and reach test | 0.10 | -0.27 to 0.47 [51] | 0.39 | | -0.30 to 1.10 [56] | |
| Speed or agility | response time | 0.19  0.46 | -0.56 to 0.18 [51]  -0.38 to 1.31 [49] |  | |  | |
|  | side step test | 0.01  0.55 | -0.36 to 0.38 [51]  -0.12 to 1.23 [60] |  | |  | |
| Balance | closed-eye foot balance | -0.18 | -0.56 to 0.19 [51] |  | |  | |
|  | right leg stance medial | 0.66 | -0.03 to 1.34 [60] |  | |  | |
|  | right leg stance posterior | 0.57 | -0.11 to 1.25 [60] |  | |  | |
|  | left leg stance posterior | 0.59 | -0.09 to 1.27 [60] |  | |  | |
|  | left-leg stance medial | 0.47 | -0.21 to 1.14 [60] |  | |  | |
|  | range of ML COP displacements |  |  | 0.69  0.15 | | 0.08 to 1.30* [44]  -0.60 to 0.89 [58] | |
|  | SD of ML COP displacements |  |  | 0.61 | | 0.04 to 1.22* [44] | |
|  | range of AP COP displacement |  |  | 0.42  0.15 | | -0.18 to 1.02 [44]  -0.79 to 0.67 [58] | |
|  | SD of AP COP displacement |  |  | 0.32 | | -0.30 to 0.91 [44] | |
|  | COP velocity |  |  | 0.46 | | -0.14 to 1.06 [44] | |
|  | Timed Up and Go Test |  |  | 1.44 | | 0.76 to 2.12* [59] | |
|  | Flamingo Balance Test |  |  | 0.20 | | -0.41 to 0.80 [59] | |
|  | Static balance – right leg | 1,28 | 0.36 to 2.21 [49] * |  | |  | |
|  | Static balance – left leg | 0,26 | -0.58 to 1.10 [49] |  | |  | |
|  | Static balance – double leg | 0,62 | -0.24 to 1.48 [49] |  | |  | |
|  | Dynamic balance – double leg | 0,67 | -0.19 to 1.53 [49] |  | |  | |
| Skills | average shooting score | 1.95 | 1.38 to 2.52 [47] * |  | |  | |
|  | golf putt performance | 2.25 | 1.04 to 3.46 [50] | -0.79 | | -1.50 to 0.18 [50] | |
| Player Experience | rates of perceived exertion |  |  | 0.80 | | 0.18 to 1.42 [44] | |
|  | autotelic experience |  |  | 0.20 | | -0.39 to 0.80 [44] | |
|  | clear goals |  |  | 0.28 | | -0.32 to 0.87 [44] | |
|  | unambiguous feedback |  |  | 0.30 | | -0.29 to 0.90 [44] | |
|  | action-awareness merging |  |  | 0.44 | | -0.15 to 1.04 [44] | |
|  | transformation of time |  |  | 0.38 | | -0.22 to 0.97 [44] | |
|  | challenge-skill balance |  |  | 0.17 | | -0.42 to 0.76 [44] | |
|  | concentration of tasks |  |  | 0.18 | | -0.41 to 0.78 [44] | |
|  | paradox of control |  |  | 0.15 | | -0.45 to 0.74 [44] | |
|  | loss of self-conscious |  |  | 0.06 | | -0.65 to 0.53 [44] | |
|  | Autonomy |  |  | 0.12 | | -0.47 to 0.72 [52] | |
|  | relatedness |  |  | -0.15 | | -7.44 to 0.45 [52] | |
|  | competence |  |  | -0.12 | | -0.71 to 0.48 [52] | |
|  | exercise enjoyment |  |  | -0.15 | | -0.76 to 0.44 [52] | |
| Adverse events |  | no adverse events occurred [45, 56] | | |  |  | |
| PA level | sedentary | SDT-off: 0.37  SDT-on: 0.56  SDT-pass: 0.28 | -0.21 to 0.96 [57]  -0.02 to 1.13 [57]  -0.33 to 0.88 [57] |  | |  | |
|  | light PA | SDT-off: 0.00  SDT-off: 0.18 | -0.58 to 0.58 [57]  -0.38 to 0.75 [57] |  | |  | |
|  | MPA | 0.13 | -0.91 to 0.64 [45] |  | |  | |
|  | MVPA | -0.01  SDT-off: 0.37  SDT-on: 0.85 | -0.77 to 0.74 [45]  -0.21 to 0.96 [57]  0.25 to 1.44 [57] * |  | |  | |
|  | VPA | -0.24 | -1.00 to 0.52 [45] |  | |  | |
|  | VVPA | 0.75 | -0.03 to 1.53 [45] |  | |  | |
|  | Freedson Adult  Freedson VM3  Troiano |  |  | 0.03  -0.07  0.11 | | -0.57 to 0.63 [52]  -0.66 to 0.53 [52]  -0.49 to 0.71 [52] | |
| Attitudes | social influence |  |  | 0.83 | | 0.21 to 1.44 [44] * | |
|  | behavioral intention |  |  | 0.95 | | 0.33 to 1.58 [44] * | |
|  | performance expectancy |  |  | 0.73 | | 0.12 to 1.35 [44] * | |
|  | effort expectancy |  |  | 0.59 | | -0.02 to 1.19 [44] | |
|  | facilitating conditions |  |  | 0.47 | | -0.13 to 1.07 [44] | |
|  | intrinsic motivation |  |  | 0.13 | | -0.47 to 0.73 [52] | |
|  | identified regulation |  |  | -0.14 | | -0.73 to 0.46 [52] | |
|  | introjected regulation |  |  | -0.12 | | -0.71 to 0.48 [52] | |
|  | extrinsic regulation |  |  | 0.08 | | -0.51 to 0.68 [52] | |
|  | amotivation |  |  | 0.06 | | -0.54 to 0.66 [52] | |
|  | Mental well-being | 1.19 | 0.28 to 2.10 [49] * |  | |  | |
